# Characterization of a New M4 Metalloprotease With Collagen-Swelling Ability From Marine *Vibrio pomeroyi* Strain 12613

**DOI:** 10.3389/fmicb.2020.01868

**Published:** 2020-08-07

**Authors:** Yan Wang, Bai-Xue Liu, Jun-Hui Cheng, Hai-Nan Su, He-Min Sun, Chun-Yang Li, Liuyan Yang, Qing-Tao Shen, Yu-Zhong Zhang, Xia Zhang, Xiu-Lan Chen

**Affiliations:** ^1^State Key Laboratory of Microbial Technology, Marine Biotechnology Research Center, Shandong University, Qingdao, China; ^2^College of Marine Life Sciences, Frontiers Science Center for Deep Ocean Multispheres and Earth System, Ocean University of China, Qingdao, China; ^3^Laboratory for Marine Biology and Biotechnology, Qingdao National Laboratory for Marine Science and Technology, Qingdao, China; ^4^School of Life Science and Technology, iHuman Institute, ShanghaiTech University, Shanghai, China; ^5^Shanghai Institute of Biochemistry and Cell Biology, University of Chinese Academy of Sciences, Shanghai, China; ^6^Department of Molecular Biology, Qingdao Vland Biotech Inc., Qingdao, China

**Keywords:** marine microbial protease, the thermolysin family (M4), collagen swelling, proteoglycans, glycoproteins

## Abstract

The ocean harbors a variety of bacteria that contain huge protease resources and offer a great potential for industrial and biotechnological applications. Here, we isolated a protease-secreting bacterium *Vibrio pomeroyi* strain 12613 from Atlantic seawater and purified a protease VP9 from strain 12613. VP9 was identified as a metalloprotease of the M4 family. VP9 could hydrolyze casein and gelatin but not elastin and collagen. With gelatin as the substrate, VP9 showed the highest activity at 40°C and pH 6.0–8.0. It was stable at temperatures of 50°C and less and in the range of pH 5.0–11.0. VP9 also had good tolerance to NaCl, non-ionic detergents, and organic solvent methanol. Unlike other M4 metalloproteases, VP9 has distinct collagen-swelling ability, and its collagen-swelling effect was concentration dependent. The relative expansion volume of collagen increased by approximately eightfold after treatment with 10 μM VP9 at 37°C for 12 h. The collagen-swelling mechanism of VP9 on bovine-insoluble type I collagen was further studied. Atomic force microscopy observation and biochemical analyses showed that VP9 can degrade proteoglycans in collagen fibers, resulting in the release of collagen fibrils from collagen fibers and the swelling of the latter. In addition, VP9 can degrade glycoproteins, a non-collagenous constituent interacting with collagen in the skin. The characteristics of VP9, such as sufficient specificity toward proteoglycans and glycoproteins but no activity toward collagen, suggest its promising potential in the unhairing and fiber-opening processing in leather industry.

## Introduction

The ocean covers approximately 71% of the earth’s surface and contains huge and various microbial resources. Marine microorganisms play an important role in the biogeochemical cycle and are considered to be an important ecological component in the marine environment (Sowell et al., 2008). In order to adapt to marine environments (i.e., low temperature, high salt, and high pressure), marine bacteria have evolved a variety of physiological mechanisms (Qin et al., 2011). Thus, enzymes isolated from these microbes usually possess unique physiological and biochemical properties.

Proteases are enzymes that can hydrolyze proteins and peptides (Wu and Chen, 2011). Some proteases secreted by marine bacteria have been identified and characterized, most of which have special properties, indicating that marine bacteria are good sources to find novel proteases (Chen et al., 2007, 2009; Zhao et al., 2012; Ran et al., 2014). Moreover, some proteases from marine microorganisms have been shown to have promising application prospects in industry (Vázquez et al., 2008; Cristóbal et al., 2011). For example, a cold-active serine protease secreted by *Pseudoalteromonas* sp. NJ276 has potential application in low-temperature food processing (Wang et al., 2008). The S8 serine protease MCP-01 produced by marine sedimentary *Pseudoalteromonas* sp. SM9913 has high collagenolytic activity and has good potential in preparing collagen oligopeptides from cod skin (Zhao et al., 2008; Chen et al., 2017). Nevertheless, only a few proteases from marine microorganisms with biotechnical/industrial potentials have been reported. Thus, new proteases with potential biotechnological/industrial applications are still necessary to be discovered from marine microorganisms.

Collagen, the most abundant fibrous protein in mammals, is the major component of mammalian extracellular matrix (ECM). The collagen superfamily consists of 28 different types (Ricard-Blum, 2011). Type I collagen, the main component of ECM in skin, bones, and tendons (Norouzi et al., 2015), has a complex hierarchical structure. Collagen monomer (diameter, 1.5 nm) is the most basic structural unit, which are cross-linked to form microfibrils (diameter, 3.5 nm). Microfibrils accumulate to form fibrils (diameter, 50–500 nm), which further aggregate with proteoglycans to form fibers and higher structures (diameter, 50–300 μm) (Fratzl et al., 1998). Proteoglycans are involved in stabilizing interfibrillar organization of collagen fibers (Orgel et al., 2009). ECM is a complex network composed of many large molecules, in which there are many glycoproteins in addition to collagen and proteoglycans (Tanzer, 2006). Glycoproteins and proteoglycans interact with collagen to maintain the stability and affect the function of collagen (Scott, 1988). More and more attention has been paid to develop enzymes that can modify natural collagen for better biotechnology and industrial applications. For example, enzymes that can specifically modify collagen are required for the sustainable development of the leather industry. Leather processing involves several steps such as soaking, unhairing, bating, degreasing, and tanning. Enzymes, as substitutes to chemicals, have been successfully used to improve leather quality and reduce environmental pollution. Novo Nordisk developed three different enzyme preparations, Aquaderm, NUE, and Pyrase, for soaking, unhairing, and bating, respectively (Rao et al., 1998). These products are enzyme complex of microbial alkaline protease and pancreatic trypsin, which have obvious limitations owing to the non-specificity toward the non-collagenous constituents of the skin. Application of specific enzymes can reduce inappropriate opening of fiber bundles. For example, in the enzyme-based fiber-opening process, a substrate-specific enzyme, α-amylase, has been shown to disintegrate the proteoglycans and induce swelling by opening the fiber matrix (Thanikaivelan et al., 2002). Thus, enzymes with specific activity toward the non-collagenous constituents in collagen and ECM is quite useful in leather processing. However, enzymes with such specific activity have rarely been reported.

The M4 family is a large family of zinc metalloproteases in the MA(E) subclan of the MA clan of metalloproteases (Rawlings and Barrett, 2004). A large number of the M4 metalloproteases are extracellular proteases secreted by bacteria, which degrade proteins to provide nutrients for bacteria. Some metalloproteases in this family perform efficient catalysis at extreme conditions, such as at high temperatures, and in organic solvents, showing potentials for biotechnological applications (Adekoya and Sylte, 2009). Some M4 metalloproteases from bacteria have been applied in industry. For example, Thermolysin, the prototype of the M4 family, has been widely used in the synthesis of aspartame, an artificial sweetener (Ooshima et al., 1985; Erbeldinger et al., 2000; Inouye et al., 2007). Another M4 metalloprotease, Vimelysin, secreted by *Vibrio* sp. T1800, shows great potentials in peptide condensation reactions because of its high activity in organic solvents (Oda et al., 1996; Takahashi et al., 2005). In addition, some M4 metalloproteases, such as Pseudolysin from *Pseudomonas aeruginosa* and hemagglutinin from *Vibrio cholerae*, are implicated as key virulence factors in the pathogenesis of various diseases and, thus, are taken as putative targets for drug development and disease treatment (Marie-Claire et al., 1997; Benitez and Silva, 2016). For some members of family M4, their mature forms are composed of one M4 catalytic domain and one or more pre-peptidase C-terminal domains (PPC domains). For example, the mature form of the M4 metalloprotease, Vibriolysin, secreted by *Vibrio vulnificus*, retains a PPC domain in addition to the catalytic domain, which has been proven to be essential for insoluble protein degradation and erythrocyte membrane attachment (Miyoshi et al., 1997; Yun et al., 2013). Recently, the non-catalytic domain PPC (pre-peptidase C-terminal domain) from some M4 metalloproteases were reported to be able to bind and swell collagen fibers (Huang et al., 2019). However, an M4 metalloprotease that is capable of swelling collagen by specifically degrading the non-collagenous constituents has never been reported.

In this study, a protease-secreting bacterium, *Vibrio pomeroyi* strain 12613, was isolated from the Atlantic surface seawater. The most abundant protease secreted by strain 12613, named VP9, was purified and characterized. As a metalloprotease of the M4 family, VP9 can hydrolyze casein and gelatin efficiently but has almost no activity toward bovine-insoluble type I collagen. However, VP9 has strong collagen-swelling ability. Atomic force microscopy (AFM) observation shows that VP9 releases fibrils from collagen fibers, leading to collagen swelling. Biochemical analyses further suggest that VP9 swells collagen by degrading the proteoglycans that interdigitate with fibrils in collagen. In addition, VP9 can degrade glycoproteins, which are related to the structural stability of collagen and ECM. These results indicate that VP9 is a new M4 metalloprotease with specificity for the degradation of proteoglycans and glycoproteins in collagen and skin ECM, which may have a potential in the dehairing and fiber-opening processes in leather industry.

## Materials and Methods

### Collection of Seawater Samples and Screening of Protease-Producing Bacteria

Seawater samples were collected from the surface seawater at site 34°28.412′W, 31°30.308′N of the Atlantic Ocean during the second section of the Ocean’s 26th voyage in June 2012. Bacteria in the seawater samples were collected on 0.22-μm polycarbonate membranes (Millipore Co., United States) by filtration. Filtered membranes were shaken in 20 ml sterile artificial seawater containing glass beads. Gradient dilution (10^–1^–10^–6^ dilutions) was performed on each sample with sterile artificial seawater. The diluted samples were spread on solid screening medium, which was composed of 0.5% yeast extract, 0.3% casein, 1.5% agar powder, and artificial seawater (pH 8.0). The plates were then incubated at 15°C for 3–4 days until a clear hydrolytic zone around a colony was detectable. Colonies with a distinct hydrolytic zone were further purified by repeatedly streaking on screening plates. Gelatin plates were prepared by adding 2% (w/w) gelatin into a basic medium containing 0.5% yeast extract, 1.5% agar, and artificial seawater. Strains with the ability to form clear hydrolysis zone on the screening medium were further streaked on the gelatin plate and incubated at 15°C for 3–4 days to investigate whether they can form hydrolytic zones around their colonies.

### Sequencing of the 16S rRNA Gene and the Genomic DNA of Strain 12613

Strain 12613 was cultured in the medium composed of 0.5% yeast powder, 1% peptone, and artificial seawater (pH 8.0) with shaking at 15°C for 24 h. The whole genomic DNA of strain 12613 was extracted with a bacterial genomic DNA extraction kit (Omega, United States) and sequenced at Shanghai Majorbio Co., Ltd. The 16S rRNA gene of strain 12613 was amplified by PCR using the genomic DNA as template with universal primers 27F and 1492R (Lane, 1991) and then sequenced at Shanghai Biosune Biotechnology Co., Ltd. (China). The 16S rRNA gene sequence of strain 12613 was submitted to GenBank under the accession number MT228950. Strain 12613 was deposited in China Center for Type Culture Collection under the number CCTCC M 2020050.

### Purification and Identification of Protease VP9 Secreted by Strain 12613

Strain 12613 was cultured at 15°C for 60 h in the fermentation medium composed of 0.2% (w/v) yeast extract, 0.5% (w/v) gelatin, 0.3% casein, and artificial seawater (pH 8.0) with shaking. After fermentation, the culture was centrifuged at 12,000 *g* for 35 min, and the supernatant was precipitated by adding ammonium sulfate powder to a concentration of 60%. The precipitate was collected by centrifugation (12,000 *g*, 30 min) and dissolved in buffer A (50 mM Tris–HCl, pH 8.0). The sample was dialyzed in buffer A and further purified on a DEAE-Sepharose Fast Flow column (Amersham Biosciences) pre-equilibrated with the same buffer. The fractions with gelatinolytic activity were concentrated and further purified on a Sephadex G75 gel filtration column (GE Healthcare, United States) eluting with buffer B (10 mM Tris–HCl, 100 mM NaCl, pH 8.0). Protease purity was analyzed by 12.5% sodium dodecyl sulfate–polyacrylamide gel electrophoresis (SDS-PAGE). The purified protease was named VP9. The protein concentration was determined using a BCA protein assay kit (Thermo) with bovine serum albumin (BSA) as the standard.

To determine the amino acid sequence of protease VP9 and its gene sequence, the purified VP9 was subjected to nano-liquid chromatography–electrospray ionization–tandem mass spectrometry (nano-LC-ESI-MS/MS) analysis. Sample preparation and the protocol of nano-LC-ESI-MS/MS analysis were as follows. Fifty microliters of denaturing buffer (0.5 M Tris–HCl, 2.75 mM ethylene diamine tetraacetic acid, 6 M guanidine–HCl, adjusted to pH 8.1 with diluted HCl) was mixed with 10 μl of purified VP9 (≤100 μg). Then, 30 μl of 1 M dithiothreitol was added into the mixture, which was incubated at 37°C for 2 h. After incubation, 50 μl of 1 M iodoacetamide were added, and the sample was placed in the dark for 1 h. Then, the buffer in the sample was replaced with 360 μl NH_4_HCO_3_ (25 mM). Trypsin (0.5 μg/μl) was added into the sample at a ratio of 1:25 (w/w), and the sample was incubated at 37°C for 12 h. After that, the sample was desalinated using a C18 Ziptip (Millipore Co., United States) according to the method described by the manufacturer. Finally, the sample was freeze dried and dissolved in ultrapure water for nano-LC-ESI-MS/MS analysis. The result was searched in the genome annotated protein database of strain 12613 with the Proteome Discoverer software 1.4 (Thermo Scientific, United States). The N-terminal amino acid sequence of the purified VP9 was analyze by Edman degradation method with a PROCISE491 sequencer (Applied Biosystems, United States). The molecular mass of the purified VP9 was determined by ESI-MS (Bruker Impact HD Q-TOF MS). The conserved domain structure of VP9 precursor was analyzed by the Conserved Domain Database (CDD) of the National Center for Biotechnology information (NCBI) (Marchler-Bauer et al., 2007). The gene sequence encoding protease VP9 was submitted to GenBank under the accession number MT228629.

### Expression and Purification of Recombinant VP9

For overexpression and purification of the recombinant enzyme of protease VP9 (recombinant VP9), the gene sequence of VP9 without the PPC domain (M1-T507) was cloned from the genomic DNA of strain 12613 and inserted into the *Nde*I and *Xho*I sites of pET-22b(+) to construct the expression vector pET-22b-*VP9*. Then, pET-22b-*VP9* was transformed into *Escherichia coli* BL21(DE3), and the recombinant *E. coli* BL21(DE3) was cultured at 15°C for 16 h with 0.35 mM isopropyl-D-thiogalactopyranoside (IPTG) as an inducer. After cultivation, the harvested recombinant *E. coli* cells were resuspended with buffer A at 0.1 g wet cells/ml and disrupted by sonication. The recombinant VP9 was purified by affinity chromatography with nickel–nitrilotriacetic acid resin (Qiagen) and then by gel filtration chromatography on a Sephadex G75 gel filtration column (GE Healthcare, United States) eluting with buffer B. Protease purity was analyzed by 12.5% SDS-PAGE.

### Characterization of Wild-Type VP9 and Recombinant VP9

Substrate specificity of wild-type VP9 and recombinant VP9 were determined by measuring their activities toward insoluble type I collagen fibers, gelatin, casein, and elastin. Insoluble type I collagen fibers (bovine Achilles tendon) were purchased from Worthington Biochemical Corporation, United States. Gelatin, casein, and elastin were purchased from Sigma (United States). The activities toward collagen and gelatin were determined with the methods described by Li et al. (2016). One unit of enzyme activity is defined as the amount of enzyme that released 1 μmol leucine from collagen in 1 h or from gelatin in 1 min. The activities toward casein were measured at 40°C using the method of He et al. (2004). The elastinolytic activities were determined with the method described by Chen et al. (2009). With gelatin as substrate, the optimum temperature for VP9 activity was determined by measuring its activity at 0–80°C in buffer A. For the thermal-stability assay, the enzyme was incubated at different temperatures (45–70°C) for 30 min, and then, the residual activity was measured at 40°C and pH 8.0. The Britton–Robinson buffer was used to determine the optimal pH of wild-type VP9 and recombinant VP9 (Chaberek et al., 1952), which contains 0.04 M phosphoric acid, 0.04 M boric acid, and 0.04 M acetic acid, and covers a wide range of pH values from pH 3.0 to 12.0 with the adjustment of 0.2 M NaOH. The optimum pH for VP9 activity was determined by measuring its activity at 40°C in the Britton–Robinson buffers (pH 3.0–12.0). For the pH-stability assay, the enzyme was incubated at 25°C for 24 h in buffers ranging from pH 3.0 to 12.0, and then, the residual activity was measured at 40°C and pH 8.0. The effect of NaCl on VP9 activity was determined by measuring its activity in 0–4 M NaCl at 40°C and pH 8.0. For the halotolerance assay, VP9 was incubated in buffers containing different concentrations of NaCl (0–4 M) at 25°C for 24 h, and then, the residual activity was measured at 40°C and pH 8.0.

To analyze the effects of metal ions and inhibitors on VP9 activity, each ion or inhibitor was added to the reaction mixture at a final concentration of 2 mM and incubated at 4°C for 30 min. Then, the residual activity of VP9 against gelatin was measured at 40°C and pH 8.0. To analyze the effects of detergents on VP9 activity, 1% of Tween 80 (v/v), Triton X-100 (v/v), SDS (1%, w/v), and cetyl trimethyl ammonium bromide (CTAB, w/v) were added to the reaction mixture, respectively. Then, the activity of VP9 was determined at 40°C and pH 8.0. To analyze the stability of VP9 in organic solvents, the activity of VP9 was determined at 40°C and pH 8.0 in the presence of 20% (v/v) or 40% (v/v) organic solvents in the reaction mixture.

The activities of VP9 against the peptides, which are derived from type I collagen fibers and were synthesized by Qiangyao Co., Ltd. (Shanghai, China), were analyzed on high-performance liquid chromatography (HPLC). A mixture of 25 μl buffer A containing 0.05 mg/ml recombinant VP9 and 2 mg/ml synthetic peptide was incubated at 40°C for 10 h and then stopped by 1% trifluoroacetic acid (TFA). The mixture without VP9 was used as a control. The hydrolytic products were detected by using a Venusil MP C18 liquid chromatography column. The column was equilibrated with inorganic phase (0.1% TFA in ultrapure water) and eluted with a linear gradient of 1–35% organic phase (0.1% TFA in acetonitrile) in 15 min at a flowrate of 1 ml/min. The eluting peptides was monitored at 220 nm.

### Observation of the Collagen-Swelling Ability of VP9

The collagen-swelling ability of VP9 was determined with the method described by Huang et al. (2019). Five milligrams of insoluble type I collagen fibers were incubated with different concentrations (0.15, 0.5, 1.5, 5, and 10 μM) of VP9 in 2 ml buffer A at 37°C for 12 h with continuous stirring, and then, the reaction mixtures were photographed. Based on the area of the collagen fibers in the pictures, the collagen-swelling effects of VP9 was determined with ImageJ 1.46 software (NIH, United States). Collagen fibers treated with 5 μM VP9 or buffer A (control) at 37°C for 24 h were further observed by AFM. The samples were rinsed three times with distilled water. The samples were spread on the surface of freshly cleaved mica and then air dried. All AFM imaging was performed in ScanAsyst mode using a Multimode Nanoscope VIII AFM (Bruker AXS) with a J-type scanner. Cantilevers with a spring constant of 2.7 N/m was used.

### Analysis of the Collagen-Swelling Mechanism of VP9

Five milligrams of insoluble type I collagen fibers were incubated with different concentrations (5, 10, and 20 μM) of VP9 in 1 ml buffer A at 37°C with continuous stirring. The content of amino acids in the supernatant of the digested mixture released from collagen fibers was determined using a colorimetric ninhydrin method with L-leucine as the standard (Freehold, 1972). The content of the glycosaminoglycans (GAGs, a component of proteoglycan within collagen fibrils) in the supernatant of the digested mixture was determined using the dimethylmethylene blue method with chondroitin 4-sulfate as the standard (Farndale et al., 1986). Collagen fibers incubated with buffer A served as the control. Decorin (0.5 mg) and fibronectin (0.5 mg) purchased from Sigma (United States) were incubated with 0.5 μM VP9 in 200 μl buffer A at 37°C for 0, 5, 10, or 20 min, and the hydrolytic products were analyzed by SDS-PAGE.

## Results

### Isolation and Identification of the Protease-Producing Bacterium *Vibrio pomeroyi* Strain 12613 From the Atlantic Surface Seawater

**FIGURE 1:**
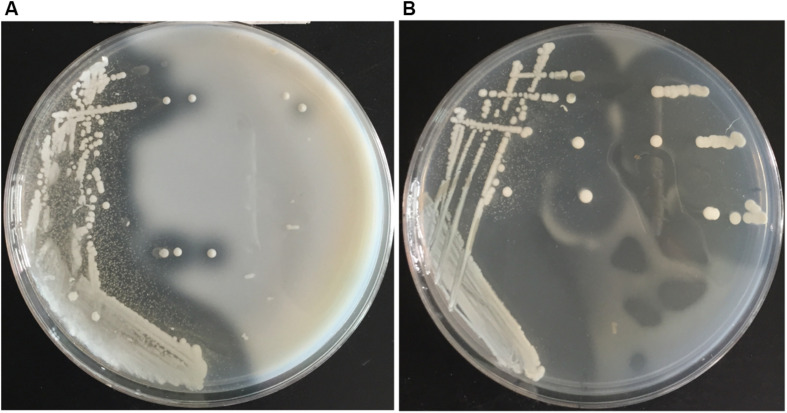
The hydrolytic zones formed by strain 12613 growing on the **(A)** plate containing casein and the **(B)** plate containing gelatin after incubation at 15°C for 3 days.

Screening plates containing casein was used to isolate protease-producing bacteria from the Atlantic surface seawater sample. A number of colonies appeared on the screening plates after cultivation at 15°C for 3–4 days, and approximately 50% colonies produced a clear hydrolytic zone, suggesting that these isolates could secrete proteases. Totally, 106 protease-producing isolates were obtained. Among them, 95 isolates could grow on the gelatin plates and form a clear hydrolytic zone. Among these isolates, the isolate 12613 produced an obvious hydrolytic zone on the screening plate containing casein (Figure 1A) and the largest hydrolytic zone on the gelatin plate (Figure 1B). This isolate was selected for further study.

To identify the isolate 12613, the 16S rRNA gene was cloned from its genome DNA and sequenced. The 16S rRNA gene sequence of the isolate 12613 shared 100% identity with *V. pomeroyi* LMG 20537^T^ (AJ491290). Thus, we named the isolate 12613 as *V. pomeroyi* strain 12613 (hereafter strain 12613).

### Purification and Identification of Protease VP9 Secreted by Strain 12613

**FIGURE 2:**
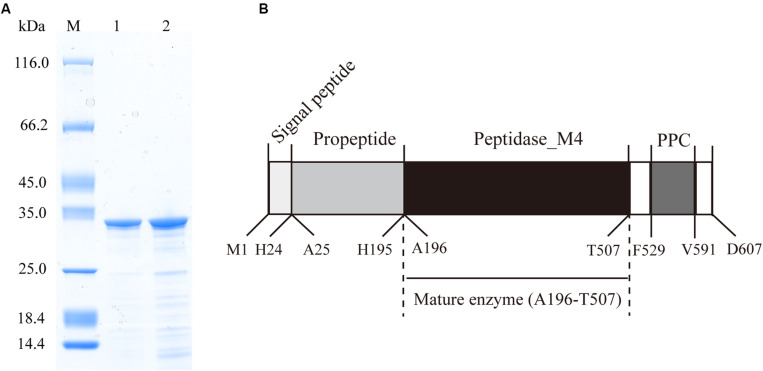
Sodium dodecyl sulfate–polyacrylamide gel electrophoresis (SDS-PAGE) analysis of the purified VP9 and schematic diagram of the conserved domain structure of VP9 precursor. **(A)** SDS-PAGE analysis of wild-type VP9 and recombinant VP9. Lane M, protein molecular mass marker; lane 1, the purified wild-type VP9; lane 2, the purified recombinant VP9. **(B)** Schematic diagram of the conserved domain structure of VP9 precursor.

The protease secreted by strain 12613 was purified from the culture of strain 12613 by ammonium sulfate precipitation and ion-exchange chromatography. SDS-PAGE analysis showed that the purified protease has an apparent molecular mass of approximately 34 kDa (Figure 2A, lane 1), which was named protease VP9 (wild-type VP9) in this study.

To identify protease VP9 and its gene, the genome DNA of strain 12613 was sequenced, and the genome annotated protein database was obtained. The purified protease VP9 was analyzed by nano-LC-ESI-MS/MS, and the result was searched in the protein database of strain 12613. Based on the result of protein database search, the complete amino acid sequence of protease VP9 was determined, and then, the gene encoding protease VP9 was identified from the annotated genome of strain 12613. The gene of VP9 contains 1,821 bp and encodes a protein of 607 amino acid residues with a calculated molecular mass of 65.92 kDa, which is the precursor of VP9.

The VP9 precursor shares 96.87% similarity with the M4 protease Vimelysin from *Vibrio* sp. T1800 and 70.87% similarity with the M4 protease Vibriolysin from *Vibrio proteolyticus*, suggesting that VP9 is a member of the M4 family. According to BLAST analysis against Conserved Domain Database, the precursor of VP9 contains an N-terminal propeptide (M1-H195), an M4 catalytic domain (A196-T507), and a PPC domain (F529-V591) (Figure 2B). The precursor of VP9 contains a putative signal peptide (M1-H24) predicted by signalP (Bendtsen et al., 2004). The N-terminal sequence of purified VP9 was determined to be AKSSGTGPGG. Mass spectrometry analysis indicated that the molecular mass of purified VP9 was 33.99 kDa. Based on its molecular mass and N-terminal sequence, it was determined that mature VP9 contains 312 amino acid residues (A196-T507). Both the N-terminal propeptide and the C-terminal PPC domain in the precursor are cleaved off during enzyme maturation, similar to many other M4 proteases (O’Donohue and Beaumont, 1996; Gao et al., 2010; He et al., 2012). The mature VP9 contains only the M4 catalytic domain.

### Characterization of the Purified Wild-Type VP9 and Recombinant VP9

**FIGURE 3:**
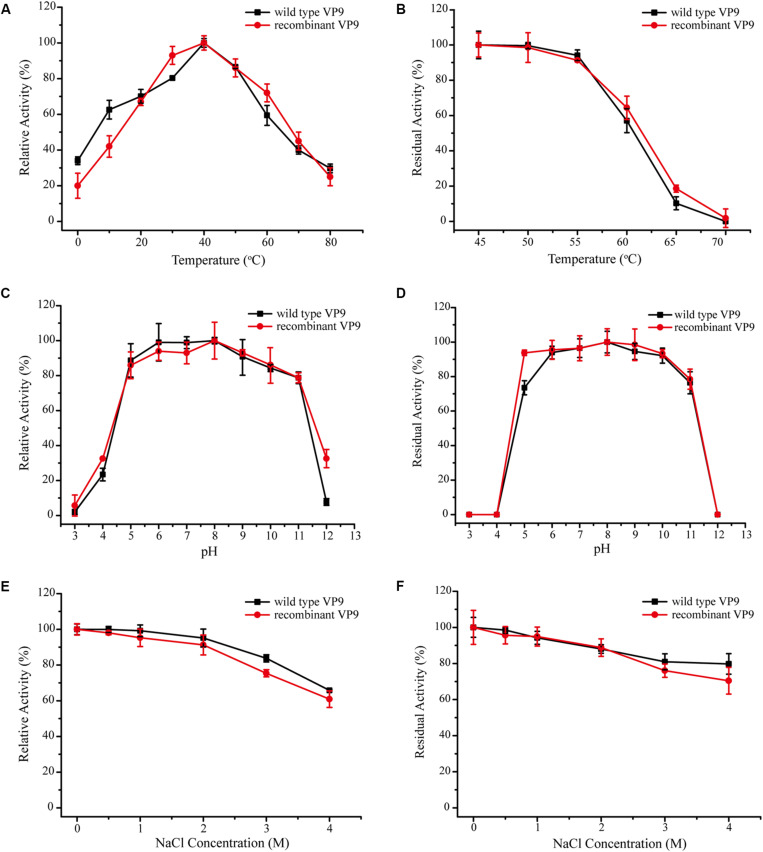
Characterization of wild-type VP9 and recombinant VP9. **(A)** Effect of temperature on the gelatinolytic activity of wild-type VP9 and recombinant VP9. Enzyme activity was measured at 0–80°C in buffer A. The specific activity of wild-type VP9 and that of recombinant VP9 at 40°C were taken as 100%. **(B)** Effect of temperature on the stability of wild-type VP9 and recombinant VP9. The enzyme was incubated at different temperatures for 30 min, and then, the residual activity was measured at 40°C. The specific activity of wild-type VP9 and that of recombinant VP9 incubated at 45°C were taken as 100%. **(C)** Effect of pH on the gelatinolytic activity of wild-type VP9 and recombinant VP9. Enzyme activity was measured at 40°C in the Britton–Robinson buffers (pH 3.0–12.0). The specific activity of wild-type VP9 and that of recombinant VP9 at pH 8.0 were taken as 100%. **(D)** Effect of pH on the stability of wild-type VP9 and recombinant VP9. The enzyme was incubated at 25°C for 24 h in buffers ranging from pH 3.0 to 12.0, and then, the residual activity was measured at 40°C and pH 8.0. The specific activity of wild-type VP9 and that of recombinant VP9 incubated at pH 8.0 were taken as 100%. **(E)** Effect of NaCl concentration on the activity of wild-type VP9 and recombinant VP9. The enzyme activity was detected at 40°C in different concentrations of NaCl ranging from 0 to 4.0 M. The specific activity of wild-type VP9 and that of recombinant VP9 in 0 M NaCl were taken as 100%. **(F)** Effect of NaCl on the stability of wild-type VP9 and recombinant VP9. The enzyme was incubated in buffers containing different concentrations of NaCl (0–4 M) at 25°C for 24 h, and then, the residual activity was measured at 40°C and pH 8.0. The specific activity of wild-type VP9 and that of recombinant VP9 incubated with 0 M NaCl were taken as 100%. The graph shows data from triplicate experiments (mean ± SD).

In addition to purifying protease VP9 (wild-type VP9) from strain 12613, we also expressed protease VP9 in *E. coli* BL21(DE3) and purified the recombinant enzyme of protease VP9 (recombinant VP9). The recombinant VP9 was purified from cell-free extracts by affinity chromatography and gel filtration chromatography, with a yield of 3.5 mg/g wet cell paste, which is higher than that (2.9 mg/g wet cell paste) of the recombinant Vimelysin (Takahashi et al., 2005). Then, we characterized both wild-type VP9 and recombinant VP9. SDS-PAGE analysis showed that recombinant VP9 has a similar molecular mass as wild-type VP9, suggesting that it is a mature enzyme (Figure 2A, lane 2). With gelatin as substrate, wild-type VP9 and recombinant VP9 showed the same optimum temperature of 40°C (Figure 3A). The activity of both enzymes showed no significant loss when pre-incubation at temperatures below 50°C for 30 min but gradually decreased when the pre-incubation temperatures were more than 50°C until completely lost at 70°C (Figure 3B). Both enzymes showed high activity (≥80% of the highest activity) in a wide range of pH from 5.0 to 11.0 in the Britton–Robinson buffer, with the highest activity at pH 6.0–8.0 (Figure 3C). Both wild-type VP9 and recombinant VP9 were stable at pH 5.0–11.0, retaining more than 70% of the maximal activity at this pH range (Figure 3D). In addition, protease VP9 has good salt tolerance. The activities of both wild-type VP9 and recombinant VP9 decreased slowly with the increase in NaCl and still had 60% activity in 4 M NaCl (Figure 3E). Moreover, both enzymes retained 70% activity after incubation in 4 M NaCl for 24 h (Figure 3F). The salt-tolerant property of VP9 reflects its adaptation to marine salty environment.

Effects of metal ions on the gelatinolytic activities of wild-type VP9 and recombinant VP9 are shown in Table 1. The activities of wild-type VP9 and recombinant VP9 were completely inhibited by 2 mM Zn^2+^, and 2 mM Cu^2+^ or Ni^2+^ severely inhibited the enzyme activities by more than 50%. Effects of inhibitors and detergents on the gelatinolytic activities of wild-type VP9 and recombinant VP9 are shown in Table 2. The activities of wild-type VP9 and recombinant VP9 were almost completely inhibited by 2 mM *o*-P (*o*-phenanthroline) and were partly inhibited by 2 mM ethylene diamine tetraacetic acid (EDTA) and ethylene glycol tetraacetic acid (EGTA), supporting that VP9 is a metalloprotease. In the presence of 1% non-ionic detergents Tween 80 and Triton X-100, both wild-type VP9 and recombinant VP9 retained more than 95 and 55% activities, respectively, but both were inactivated by 1% ionic detergent CTAB or SDS. In addition, both wild-type VP9 and recombinant VP9 showed good tolerance to several organic solvents at a concentration of 20%, including acetonitrile, methanol, and ethanol (Table 3). Notably, both enzymes retained more than 80% activity in 40% methanol (Table 3).

**TABLE 1:** Effects of metal ions on the gelatinolytic activities of wild-type VP9 and recombinant VP9.

**Metal ion**	**Relative activity^a^ (%)**		**Metal ion**	**Relative activity^a^ (%)**	
	**Wild-type**	**Recombinant**		**Wild-type**	**Recombinant**
	**VP9**	**VP9**		**VP9**	**VP9**
Li^+^	95.75 ± 1.29	85.17 ± 3.39	Ca^2+^	69.96 ± 2.13	81.54 ± 2.44
K^+^	93.24 ± 4.53	94.48 ± 2.27	Fe^2+^	43.06 ± 4.48	63.67 ± 3.11
Co^2+^	91.98 ± 0.96	71.98 ± 2.11	Ni^2+^	33.78 ± 3.28	49.30 ± 3.92
Mg^2+^	80.18 ± 3.16	96.39 ± 1.40	Cu^2+^	26.86 ± 1.26	26.04 ± 4.34
Ba^2+^	78.61 ± 3.00	83.67 ± 3.26	Zn^2+^	0.00 ± 0.00	0.00 ± 0.00
Mn^2+^	73.26 ± 4.58	89.82 ± 1.31			

**^*a*^*The activity of VP9 toward gelatin was measured at 40°C and pH 8.0. The specific activity of wild-type VP9 and that of recombinant VP9 without any metal ions were taken as 100%. The data shown in the table are from triplicate experiments (mean ± SD).*

**TABLE 2:** Effects of inhibitors and detergents on the gelatinolytic activities of wild-type VP9 and recombinant VP9.

**Inhibitor^*a*^**	**Residual activity^*b*^ (%)**		**Detergent^*c*^**	**Relative activity^*d*^ (%)**	
	**Wild-type**	**Recombinant**		**Wild-type**	**Recombinant**
	**VP9**	**VP9**		**VP9**	**VP9**
PMSF	94.36 ± 5.41	92.34 ± 1.64	Tween 80	96.65 ± 4.35	98.97 ± 4.01
EGTA	78.10 ± 1.30	60.45 ± 1.50	Triton X-100	56.44 ± 4.65	64.98 ± 7.40
EDTA	70.73 ± 0.65	68.18 ± 2.62	SDS	0.00 ± 0.00	2.08 ± 0.57
*o*-P	4.74 ± 1.57	5.67 ± 1.77	CTAB	0.00 ± 0.00	0.00 ± 0.00

**^*a*^*PMSF, p-amidinophenylmethylsulfonyl fluoride; EDTA, ethylene diamine tetraacetic acid; EGTA, ethylene glycol tetraacetic acid; *o*-P, *o*-phenanthroline. *^*b*^*VP9 was incubated with each inhibitor at 4°C for 30 min, and the residual activity against gelatin was measured at 40°C and pH 8.0. The specific activity of wild-type VP9 and that of recombinant VP9 without any inhibitor were taken as 100%. The data shown in the table are from triplicate experiments (mean ± SD). *^*c*^*SDS, sodium dodecyl sulfate; CTAB, cetyl trimethyl ammonium bromide. *^*d*^*The activity of VP9 toward gelatin was measured in buffer A containing 1% detergent at 40°C and pH 8.0. The specific activity of wild-type VP9 and that of recombinant VP9 without the addition of detergent were taken as 100%. The data shown in the table are from triplicate experiments (mean ± SD).*

**TABLE 3:** Effects of organic solvents on the gelatinolytic activities of wild-type VP9 and recombinant VP9.

**Organic solvent 20% (v/v)**	**Relative activity^*a*^ (%)**		**Organic solvent 40% (v/v)**	**Relative activity^*a*^ (%)**	
	**Wild-type VP9**	**Recombinant VP9**		**Wild-type VP9**	**Recombinant VP9**
Acetonitrile	96.10 ± 6.23	91.22 ± 4.50	Acetonitrile	47.93 ± 6.85	37.74 ± 4.24
Methanol	86.05 ± 1.43	87.93 ± 8.69	Methanol	88.33 ± 7.07	83.90 ± 0.03
Ethanol	76.02 ± 3.02	79.80 ± 6.28	Ethanol	69.02 ± 0.62	43.80 ± 0.65
Acetone	68.69 ± 6.13	71.10 ± 1.47	Acetone	39.85 ± 7.19	45.27 ± 1.75
DMSO*^*b*^*	62.76 ± 1.80	71.80 ± 3.75	DMSO*^*b*^*	46.14 ± 4.60	46.77 ± 1.49
Isopropanol	61.49 ± 1.38	58.78 ± 6.20	Isopropanol	19.55 ± 7.76	19.56 ± 4.41
n-Propanol	43.77 ± 7.00	47.57 ± 3.35	n-Propanol	0.00 ± 0.00	0.00 ± 0.00

**^*a*^*The activity of VP9 toward gelatin was determined in the presence of 20 or 40% organic solvents at 40°C and pH 8.0. The specific activity of wild-type VP9 and that of recombinant VP9 with the addition of equal volume of water instead of an organic solvent in the reaction mixture were taken as 100%. The data shown in the table are from triplicate experiments (mean ± SD). *^*b*^*DMSO, dimethyl sulfoxide.*

**FIGURE 4:**
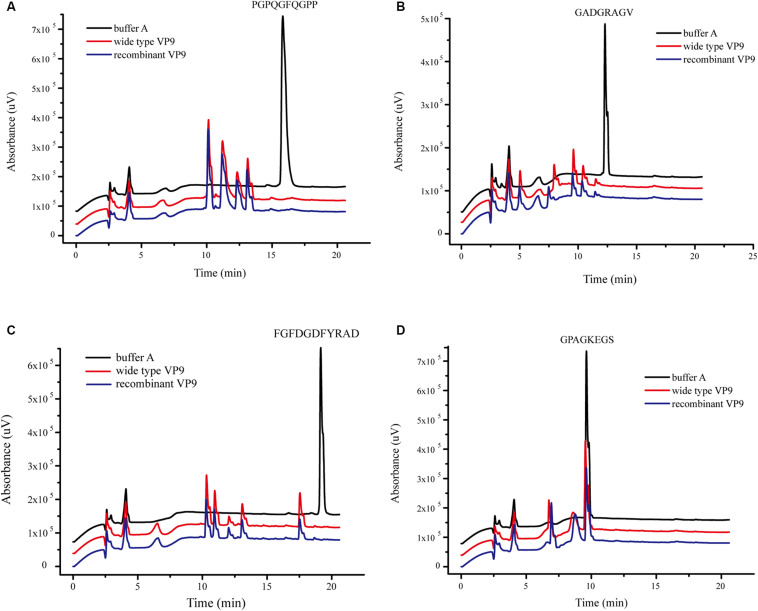
The degradation of VP9 on synthetic peptides derived from type I collagen detected with high-performance liquid chromatography (HPLC). Black line, the synthetic peptides were incubated in 20 μl buffer A without VP9 for 10 h at 40°C, which served as the control. Red and blue lines, the synthetic peptides were treated with 0.05 mg/ml wild-type VP9 (red line) or recombinant VP9 (blue line) in 20 μl buffer A for 10 h at 40°C. **(A)** Peptide PGPQGFQGPP, **(B)** peptide GADGRAGV, **(C)** peptide FGFDGDFYRAD, and **(D)** peptide GPAGKEGS.

The substrate specificity of protease VP9 to various proteinaceous substrates is shown in Table 4. Both wild-type VP9 and recombinant VP9 had activity toward casein but neither had activity toward elastin. Although both wild-type VP9 and recombinant VP9 could hydrolyze gelatin, neither could hydrolyze bovine-insoluble type I collagen fibers. However, we found that VP9 could hydrolyze synthetic peptides derived from type I collagen (Figure 4), consistent with the observation that VP9 had activity against gelatin, the heat-denatured form of collagen (Table 4).

**TABLE 4:** The substrate specificity of wild-type VP9 and recombinant VP9.

**Substrate**	**Activity^*a*^ (U/mg)**	
	**Wild-type VP9**	**Recombinant VP9**
Bovine-insoluble type I collagen fiber	0.27 ± 0.01	0.46 ± 0.01
Gelatin	61.88 ± 4.81	68.43 ± 4.67
Casein	1453.71 ± 4.10	1147.0 ± 13.23
Elastin-orcein	ND	1.57 ± 0.18

**^*a*^*The specific activity of VP9 toward each substrate was measured at 40°C and pH 8.0. The data shown in the table are from triplicate experiments (mean ± SD). ND means that the enzyme activity was not detectable.*

Taken together, the results showed that there are no significant differences in the characteristics between wild-type VP9 and recombinant VP9. Thus, recombinant VP9 was used for further study.

### Observation of the Collagen-Swelling Ability of Protease VP9

**FIGURE 5:**
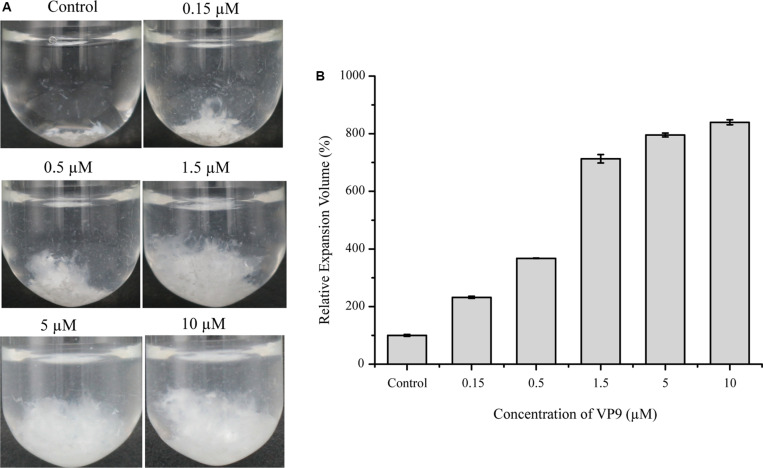
The collagen-swelling effect of VP9. **(A)** Insoluble type I collagen fibers treated with different concentrations of VP9. Insoluble type I collagen fibers (5 mg) in 2 ml buffer A containing different concentrations of VP9 (0.15, 0.5, 1.5, 5, and 10 μM) were incubated at 37°C for 12 h. Collagen incubated with 2 ml buffer A containing no VP9 was used as a control. **(B)** Statistical analysis of the relative expansion volumes of collagen fibers treated with different concentrations of VP9 (0.15, 0.5, 1.5, 5, and 10 μM) at 37°C for 12 h. The volume of collagen fibers treated with buffer A was taken as 100%. Values are expressed as mean ± SD from three measurements.

Although VP9 had almost no activity on bovine-insoluble collagen fibers, we found that VP9 had distinct collagen-swelling ability. When incubating with VP9 at 37°C for 12 h, the compact bovine-insoluble type I collagen fibers became loose to a large extent, which seemly depended on VP9 concentration (Figure 5A). We measured the change in the volume of collagen treated with different concentrations of VP9, and the results are shown in Figure 5B. With the increase in the concentration of VP9, the relative expansion volume of the VP9-treated collagen increased. The relative expansion volume of the collagen treated with 10 μM VP9 increased by approximately eightfold compared with the collagen treated with buffer A under the same conditions.

**FIGURE 6:**
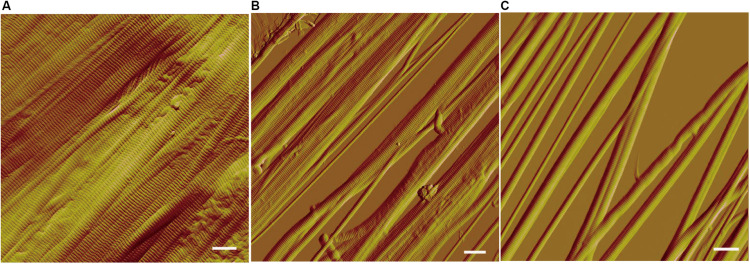
Atomic force microscopy (AFM) observation of the collagen fibers treated with 5 μM VP9 for 24 h. **(A)** Collagen fibers treated with buffer A. **(B,C)** Collagen fibers treated with VP9. Packed fibril bundles in the collagen fibers were dissociated into free fibril bundles and single fibrils by VP9. Bars, 1 μm.

We further observed the collagen fibers treated with VP9 under AFM. AFM observation showed that collagen fibrils were closely packed within the collagen fibers treated with buffer A (Figure 6A). However, after VP9 treatment, packed fibril bundles in the collagen fibers were dissociated into free fibril bundles and even fibrils (diameter, 100 nm to 1 μm) (Figures 6B,C). These results indicate that VP9 swelled collagen via dissociating the collagen fibrils in collagen fibers.

### Analysis of the Collagen-Swelling Mechanism of VP9

**FIGURE 7:**
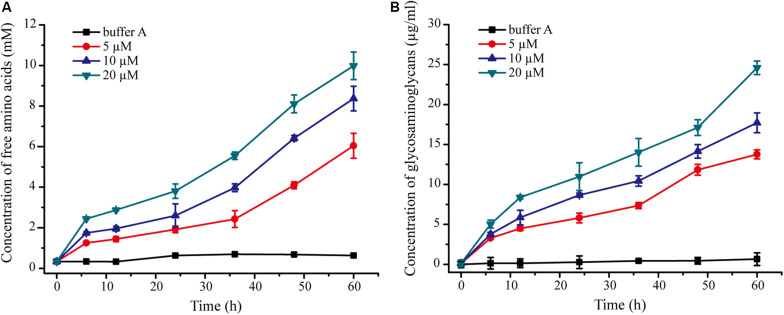
Detection of amino acids and glycosaminoglycans (GAGs) released from collagen fibers by VP9 treatment. **(A)** Amino acids released from collagen fibers by VP9. **(B)** GAGs released from collagen fibers by VP9. Collagen fibers incubated with buffer A were used as controls. The graph shows data from triplicate experiments (mean ± SD).

**FIGURE 8:**
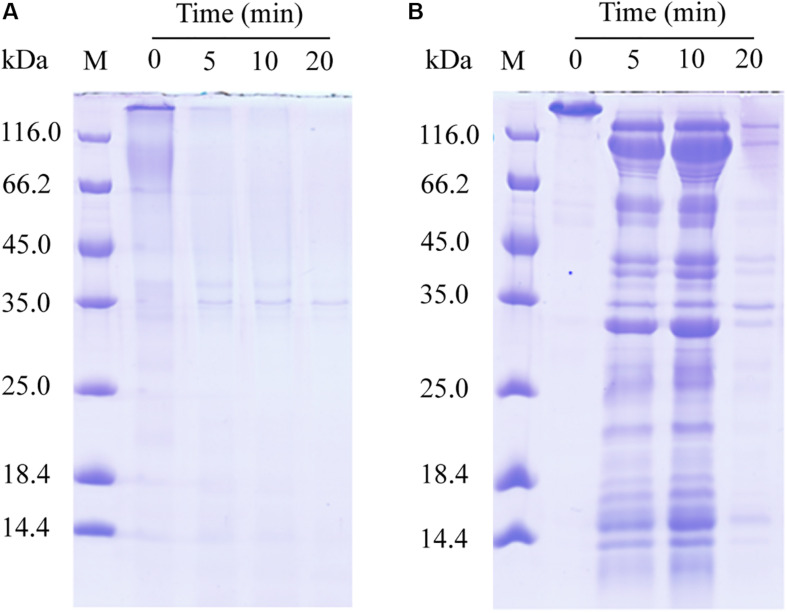
Sodium dodecyl sulfate–polyacrylamide gel electrophoresis (SDS-PAGE) analysis of the hydrolysis of decorin and fibronectin by VP9. **(A)** The hydrolysis of decorin by VP9. Lane M, protein molecular mass marker. Lanes 0, 5, 10, and 20, decorin treated with 0.5 μM VP9 in 200 μl buffer A for 0, 5, 10, and 20 min, respectively. **(B)** The hydrolysis of fibronectin by VP9. Lane M, protein molecular mass marker. Lanes 0, 5, 10, and 20, fibronectin treated with 0.5 μM VP9 in 200 μl buffer A for 0, 5, 10, and 20 min, respectively.

We further probed the molecular mechanism of VP9 to swell collagen fibers by biochemical analyses. The dispersion of collagen fibrils from the collagen fibers treated with VP9 suggests that substances that are responsible for stabilizing interfibrillar organization in collagen fibers may be degraded by VP9. To confirm this, a series of biochemical experiments were performed. Proteoglycans, which consist of core proteins and GAGs, are involved in stabilizing the interfibrillar organization of collagen fibers, as they are the most important interfibrillary proteins in collagen fibers (Dettmer et al., 2012). If core proteins in collagen fibers are degraded by VP9, amino acids and GAGs would be released from the collagen fibers. To test this, collagen fibers were treated with VP9, and released amino acids and GAGs in the reaction mixture were detected. As expected, both amino acids (Figure 7A) and GAGs (Figure 7B) were detected in the supernatant of the reaction mixture, the amount of which increased with both treatment time and VP9 concentration. Moreover, SDS-PAGE analysis clearly showed that decorin, the archetypal proteoglycan in collagen fibers, could be digested by VP9 (Figure 8A). These results indicate that the proteoglycans within collagen fibers can be degraded by VP9.

Fibronectin is a large glycoprotein that binds to collagen and other proteoglycans to form the ECM network (Ruoslahti et al., 1982), playing an important role in regulating the structure of type I collagen and determining the stability of ECM (Sottile et al., 2007). We investigated the activity of VP9 against fibronectin by SDS-PAGE. The result clearly showed that fibronectin can be hydrolyzed into peptides by VP9 (Figure 8B).

Altogether, these results indicate that VP9 dissociates collagen fibers into collagen fibrils by digesting the interfibrillary proteoglycans, resulting in collagen swelling. In addition, VP9 can degrade glycoproteins, thereby affecting the structural stability of collagen in the skin ECM network.

## Discussion

In this study, we isolated a protease-secreting bacterial strain 12613 from the Atlantic surface seawater, which was identified as a strain of *V. pomeroyi.* The 16S rRNA gene sequence of *V. pomeroyi* strain 12613 shares 100% identity with the type strain *V. pomeroyi* LMG 20537^T^ (AJ491290). Strain LMG 20537^T^ was isolated from bivalve (*Nodipecten nodosus*) larvae in southern Brazil (Thompson et al., 2003). It has not been reported whether strain LMG 20537^T^ has protease-secreting capacity. Here, we found that strain 12613 had the biggest protease-secreting capacity among the isolated strains. Therefore, we further purified and characterized the protease secreted by strain 12613.

We purified a protease, VP9, from strain 12613. VP9 was identified as an M4 metalloprotease based on genome sequencing of strain 12613 and nano-LC-ESI-MS/MS analysis of purified VP9. As an M4 metalloprotease, VP9 shares 96.87% similarity with Vimelysin and 70.87% similarity with Vibriolysin, two representative members of M4 metalloproteases. Vimelysin can degrade casein and furylacryloyl-glycyl-leucine amide (FAGLA) (Oda et al., 1996), while Vibriolysin can degrade casein, gelatin, and cross-linked fibrin such as fibrinogen, fibrin, and Factor Xa (Kwon et al., 2007). Similarly, we found that VP9 shows high activity against casein and gelatin. In addition, we found that VP9 has a strong ability to swell insoluble collagen fibers but no activity against insoluble collagen fibers. It has been reported that the average volume of collagen treated with 0.15 μM of the PPC domains from several bacterial proteases increased by approximately fivefold (Huang et al., 2019). At this protein concentration, the volume of VP9-treated collagen increased by approximately 2.3-fold, indicating that the collagen-swelling effect of VP9 is weaker than that of the PPC domains from bacterial proteases. However, we found that the collagen-swelling effect of VP9 is concentration dependent. The relative expansion volume of the collagen treated with 10 μM VP9 increased by approximately eightfold. Moreover, VP9 has good thermostability and is stable in a range of pH 5.0–11.0. It also has good tolerance to NaCl, non-ionic detergents, and some organic solvents. These properties indicate its potential for industrial application.

Because protease with collagen-swelling ability may have potentials in industry, such as in leather processing, we further probed the collagen-swelling mechanism of VP9 by AFM observation and biochemical analyses. Our results showed that VP9 can degrade proteoglycans to dissociate collagen fibril bundles and fibrils from collagen fibers, thereby leading to collagen swelling. In addition, VP9 also had the ability to degrade glycoproteins, which are essential components of ECM. It has been reported that enzymes assisting the removal of proteoglycans contribute to the opening up of fiber bundles to soften the leather (Dettmer et al., 2012). Glycoproteins are essential for holding the hair follicle in skin ECM, and thus, the degradation of glycoproteins would contribute to the complete dislodging of tiny skin hair in leather dehairing processing (Sivasubramanian et al., 2008). Therefore, enzymes with specific activity toward proteoglycans and glycoproteins are useful in dehairing and fiber-opening processing. An α-amylase involved in fiber-opening method, based on its specific activity toward proteoglycans, has been shown to be feasible compared with the conventional liming processes (Thanikaivelan et al., 2002; Saravanabhavan et al., 2004). Because VP9 has specific activity toward the non-collagenous constituents, proteoglycans and glycoproteins, in ECM and no activity to the collagen fibrils, it may have promising potential in dehairing and fiber-opening processing in leather industry.

An M12 protease, myroilysin, secreted by marine *Myroides profundi* D25 was reported to have collagen-swelling ability and no collagenolytic activity (Chen et al., 2009). However, the collagen-swelling mechanism of myroilysin is still unclear. In addition, the polycystic kidney disease (PKD) domain from the S8 serine protease MCP-01 possesses the ability to bind and swell collagen fibers (Wang et al., 2010). Similar to PKD, the PPC domains from some M4 metalloproteases and S8 serine proteases also can bind and swell collagen fibers (Huang et al., 2019). However, both PKD and PPC domains in proteases are non-catalytic, which are unable to degrade either the collagenous or the non-collagenous constituents in collagen fibers. Although the key amino acid residues in the PKD and PPC domains that might be involved in binding and swelling insoluble collagen fibers have been identified (Zhao et al., 2008; Wang et al., 2010; Huang et al., 2019), the underlying mechanisms that the PKD and PPC domains swell collagen fibers are still unclear. Moreover, the PPC domains of most M4 metalloproteases are removed during enzyme maturation, and thus, mature M4 metalloproteases usually contain no PPC domain (Yeats et al., 2003). Distinct from the non-catalytic PKD and PPC domains, mature VP9 that only contains an M4 catalytic domain can swell collagen fibers by specifically degrading the non-collagenous constituents including proteoglycans and glycoproteins. Therefore, to our knowledge, VP9 may represent the first reported M4 protease that has collagen-swelling ability.

## Data Availability Statement

*Vibrio pomeroyi* strain 12613 was deposited in China Center for Type Culture Collection under the number CCTCC M 2020050. The 16S rRNA gene sequence of strain 12613 has been submitted to GenBank database under the accession number MT228950. The nucleotide sequence encoding protease VP9 has been submitted to GenBank database under the accession number MT228629.

## Author Contributions

X-LC and YW conceived and designed the research. X-LC and XZ directed the research. YW, B-XL, and J-HC conducted the experiments. H-NS and H-MS performed the AFM observation. C-YL, LY, and Q-TS helped in analyzing the data. X-LC and YW wrote the manuscript. XZ and Y-ZZ revised the manuscript. All authors read and approved the manuscript.

## Conflict of Interest

XZ was employed by Qingdao Vland Biotech Inc.

The remaining authors declare that the research was conducted in the absence of any commercial or financial relationships that could be construed as a potential conflict of interest.
